# 

**Published:** 1809-07

**Authors:** 


### MEDICAL

AND

### PHYSICAL

### JOURNAL.

CONDUCTED BY

T. BRADLEY, M.D.

AND

J. ADAMS, M. D.

VOL. XXII.

FROM JUNE TO DECEMBER, 1809.

LONDON.

PRINTED FOR RICHARD PHILLIPS, NO, 6, BRIDGE-STREET, BLACKFRIARS.

### NEW MEDICAL PUBLICATIONS.

An Inquiry into the Anti-variolous Power of Vaccination By Thomas Brown, Surgeon, Musselburgh 7s. 6d.

The English Physician enlarged. By Dr. Parkins. 5s. bound, fine paper 7s. 6d.

The Principles of Midwifery, including the Diseases of Women and Children. By John Burns, Lecturer of Midwifery, and Member of the Faculty of Physicians and Surgeons, Glasg w. 8vo. 12s.

### CORRESPONDENCE.

We have received several accounts of cow-pox failures; among the rest, a long letter from Mr. Bartlett, Apothecary to the Finsbury Dispensary. The last gives an account of Mr Taunton's, and many other children, who several years after going regularly through the process of vaccination, took the small-pox by inoculation, and some casually. The cases by inoculation of small-pox were mild, and most of the others, but not all.

We have also heard from a great friend to vaccination, that during an epidemic small-pox at Hayes, all the children formerly vaccinated, who were on this occasion inoculated to the amount of twenty, took the small-pox slightly, that is, as much as is the common effect of inoculation. Ten, who were not inoculated, resisted every exposure. It is said that some persons on this occasion had the small-pox a second time. It is of the utmost importance that all this should be rightly understood, in order to enable us to determine by what laws these diseases are governed, and how we are to arrange what at present appear anomalies. These cases should be transmitted to the National Vaccine Establishment in Leicester Square. At the same time such publicity should be allowed to every well ascertained fact, as may teach those who meet with similar incidents to mark every phenomenon in which the cases seem to differ or agree.

We are sorry that the absence of our Publisher prevented our receiving Mr. Taylor's letter till it was too late to avail ourselves of it for this Number.—It shall be attended to in the current month.

"An Essex Practitioner" cannot expect us to insert his anonymous remarks on our Correspondent who honours us with his name. If he inserts his own, confining himself to the result of his own experiments, without any unnecessary reflexions, we shall be ready to do him justice. The remedy to which he alludes does not entirely rest on the credit of the candid proposer, satisfactory as that alone might be. Besides common fame, which in matters of this kind, since the introduction of cow-pox, we shall always be afraid of despising, we have the authority of other practitioners, confirmed in one instance by our own observation. At the same time we have no intention to doubt the truth of the Essex Practitioner. The insects to which we owe the remedy are a numerous tribe, whose properties may be different, and even the same species different at different seasons, as we see most remarkably the case in all the poisonous reptiles.

Dr. SEENY's Treatise on Local Inflammation will be noticed in our next.

